# *In vitro* osteoblast activity is decreased by residues of chemicals used in the cleaning and viral inactivation process of bone allografts

**DOI:** 10.1371/journal.pone.0275480

**Published:** 2022-10-10

**Authors:** Guillaume Villatte, Roger Erivan, Stéphane Descamps, Pierre Arque, Stéphane Boisgard, Yohann Wittrant

**Affiliations:** 1 Clermont Auvergne University, CNRS, SIGMA Clermont, ICCF, Clermont–Ferrand, France; 2 Departement of Orthopaedic Surgery, CHU Montpied Clermont-Ferrand, Clermont–Ferrand, France; 3 INRAE, Clermont Auvergne University, UMR 1019 Human Nutrition, Clermont-Ferrand, France; Monash University, AUSTRALIA

## Abstract

Allograft bone tissue has a long history of use. There are two main ways of preserving allografts: by cold (freezing), or at room temperature after an additional cleaning treatment using chemicals. These chemicals are considered potentially harmful to humans. The aim of the study was (i) to assess the presence of chemical residues on processed bone allografts and (ii) to compare the *in vitro* biocompatibility of such allografts with that of frozen allografts. The presence of chemical residues on industrially chemically treated bone was assessed by high performance liquid chromatography (HPLC) after extraction. Biocompatibility analysis was performed on primary osteoblast cultures from Wistar rats grown on bone disks, either frozen (F-bone group) or treated with supercritical carbon dioxide with no added chemical (scCO2-bone group) or industrially treated with chemicals (CT-bone group). Cell viability (XTT) was measured after one week of culture. Osteoblastic differentiation was assessed after 1, 7 and 14 days of culture by measuring alkaline phosphatase (ALP) activity directly on the bone discs and indirectly on the cell mat in the vicinity of the bone discs. Residues of all the chemicals used were found in the CT-bone group. There was no significant difference in cell viability between the three bone groups. Direct and indirect ALP activities were significantly lower (−40% to −80%) in the CT-bone group after 7 and 14 days of culture (*p* < 0.05). Residues of chemical substances used in the cleaning of bone allografts cause an *in vitro* decrease in their biocompatibility. Tissue cleaning processes must be developed that limit or replace these chemicals to favor biocompatibility.

## Introduction

Allograft tissue from the human musculoskeletal system, particularly bone, has a long history of use [1]. From the 1980s onwards, the development of prosthetic joint surgery and therefore of prosthetic revisions, but also of tumor surgery of the limbs, coupled with the ease of conservation and use of tissues, meant that the use of bone allografts became common practice, [2, 3] with a continuous growth in demand [4].

There are two main ways of preserving allografts [5]. The first is cold preservation, which is the historical method of preservation and still the reference of today. Various methods are possible, but the most frequent one is direct freezing at −80°C with no added preservative. Readily available but logistically cumbersome (an unbroken cold chain is mandatory), this method of preservation has little influence on mechanical properties (about 1% change in tensile strength) [6] and reduces immunogenicity [7, 8]. The second method of preservation is at room temperature after an additional cleaning treatment, resulting in dehydration of the tissue. This additional treatment varies according to the tissue bank but is based on a combination of chemical and physical processes of ranging complexity to achieve delipidation and decellularization, finally ensuring tissue safety. The chemical treatment uses various polar protic and aprotic solvents such as ethanol, hydrogen peroxide, sodium hydroxide, acetone, chlorine, etc. Much simpler and more practical as regards storage and logistics, these treated allografts present relatively intact mechanical properties [9]. However, chemical residues left at the end of the cleaning treatment and their possible toxicities have only been scantly studied [1, 10].

The aim of this work was (i) to assess the presence of chemical residues in processed allograft bone, and (ii) to compare the *in vitro* biocompatibility of processed versus frozen allografts.

The hypothesis was that residues of the chemicals used in the additional treatment remained on treated allografts, causing a decreased biocompatibility in cell cultures.

## Materials and methods

### Ethical approval

All the human tissues came from a non-profitable tissue bank (Osteobanque, Clermont-Ferrand, France) and from an Orthopedic and Traumatology Surgery Department in a teaching Hospital. Authorization for research use was granted (DC-2021-4555).

Cells from calvaria were collected post-mortem on new born rats according to breeding procedures in our animal facility (IEN-UNH-UMR1019 / accreditation n°E-6334515).

### Origin and preservation of allograft bone

Bone tissues (cancellous and cortical) used in the F-bone group and the scCO_2_-bone group came from femurs obtained in four Multi-Organ Tissue Harvesting (MOTH) procedures. Samples used in the CT-bone group were from living female donors receiving hip arthroplasty and manufactured as demi-tete products for therapeutic use; residues (the cortical part of the neck) not used for the care of the patient, considered surgical waste, were collected for use in this study.

Cortical bone fragments were cut as discs 10 mm in diameter and 3 mm thick after the preservation procedures were carried out. They were divided into three groups according to preservation mode:

- direct freezing at −80°C with no added preservatives for minimum 3 weeks (F-bone group).
- at room temperature for 3 weeks, after an additional industrial treatment (commercially available) combining mechanical washing with water and a detergent followed by successive baths of various chemicals (propanone C_3_H_6_O, urea CH_4_N_4_), and finally dehydration with ethanol C_2_H_5_OH and low thermal drying, and a rapid Beta irradiation (CT-bone group).
- at room temperature for 3 weeks, after an additional treatment combining mechanical washing with physiological serum during one hour and then supercritical carbon dioxide treatment (scCO_2_) (at temperature of 40°C and pressure of 250 bars during 4 hours) with no added chemicals (scCO2-bone group).

### Assay of chemical residues in industrially processed allografts (chemically treated bone)

Residues of the four main chemicals used in the industrial bone processing (propanone, ethanol, urea, and detergent) were assayed by High Performance Liquid Chromatography (HPLC). An ultraviolet (UV) spectrum was run for each chemical to determine the wavelength best suited for analysis. A processed bone extract was prepared according to the Biological evaluation of medical devices: Sample preparation and reference materials ISO 10993–12:2012. The extraction was performed at 37°C with magnetic stirring for 72 h, with 10 g of CT-bone (chemically-treated bone) in 50 g of ultrapure water (corresponding to a “simulated extraction” according to the ISO).

Residues of each chemical were quantified by comparing its area under the curve (AUC) in the treated bone extract solution with that of diluted solutions of the chemical at different concentrations. HPLC measurements were made twice for each condition.

The amount of residual propanone, urea, and acetone was calculated in grams per kilogram of treated bone from the molar concentration of each substance. Since the composition of the detergent was not known, its residual amount was expressed as a dilution equivalent of the pure stock solution. The uncertainty associated with this measurement was determined, with a kurtosis coefficient of 2.21 and a skewness coefficient of 0.08 [11].

### In vitro cell cultures

#### Cell type

Primary rat osteoblastic cells were enzymatically isolated from explants obtained from fetal Wistar rat skulls as previously described [12–14]. Bone pieces were sequentially digested in a solution of α-MEM (Modified Eagle’s Medium) (Sigma-Aldrich–M6074), 1% penicillin/streptomycin (p/s), collagenase type IA (0.1%), dispase II (0.2%) at 37°C and incubated four times 15 minutes at 37°C. The rat skull cells obtained were pooled and seeded at an initial density of 10,000 cells/cm^2^ in α-MEM medium supplemented with 10% Fetal Bovine Serum (FBS) (Sigma-Aldrich–F7524) and 1% p/s in a controlled atmosphere (5% CO_2_ / 95% air, 90% humidity) at 37°C until confluence for subculturing or freezing.

### Culture of rat osteoblasts in the three bone groups

**Fig 1:**
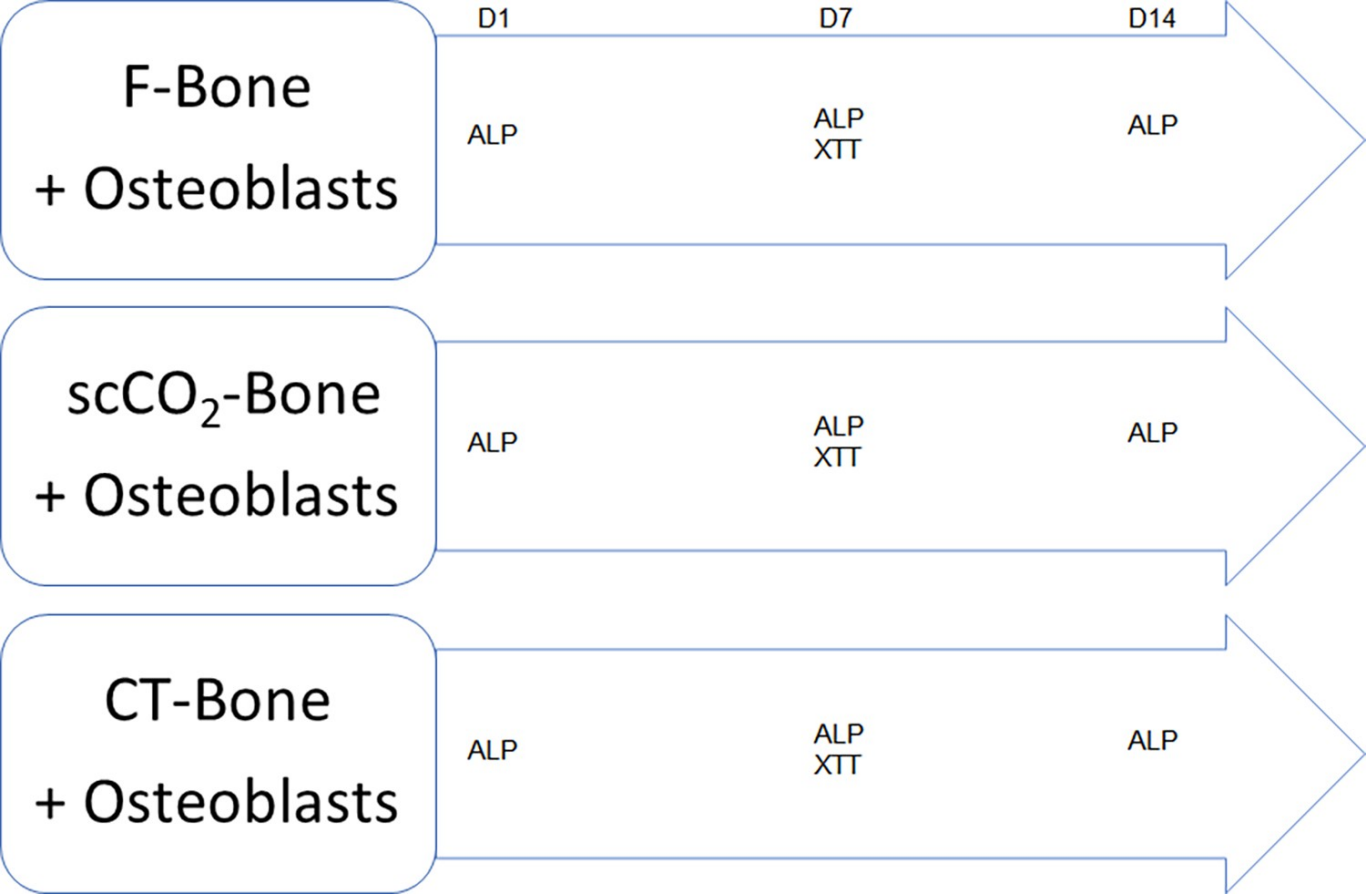
Diagram of the experimental design of osteoblast (OB) cultures on bone discs (F-Bone: Frozen-bone; scCO_2_-Bone: Non-chemically-treated bone whith supercritical CO2. CT-Bone: Chemically-treated bone. ALP: Evaluation of osteoblast differentiation and mineralization activity by alkaline phosphatase activity assay. XTT: Quantitative evaluation of cell viability by XTT (tetrazolium redox). D: Day).

Each bone disc was placed in a well of a 12-well culture plate (Falcon) and seeded with 35,000 primary osteoblasts (10,000 cells/cm^2^). The whole (bone disc + osteoblasts) was covered with 2 ml of culture medium. The plates were then incubated at 37°C and 5% CO_2_, with no shaking. The culture medium was changed three times a week, with quantification after one week (Fig 1).

#### Quantitative assessment of cell viability by the XTT method

The XTT method measures the viability of cells based on the activity of their mitochondrial enzymes, which reduce XTT (tetrazolium redox). This activity is lost when the cells die.

After one week in culture, measurements of absorbance were made using a Cell Proliferation Kit II (XTT) (Sigma-Aldrich—11465015001), with a spectrophotometric reading at a wavelength of 490 nm.

The results are presented as percentages relative to the F-bone group (100%).

The experiment was replicated 12 times for the three bone groups.

#### Evaluation of osteoblastic differentiation and mineralization activity by alkaline phosphatase (ALP) assay

ALP is considered an early marker of osteoblastic differentiation. The test is based on an enzymatic assay. Cells are lysed and placed in a buffer with pNPP (*p*-nitrophenyl phosphate), which is hydrolyzed to *p*-nitrophenol and phosphate in the presence of alkaline phosphatase.

ALP measurements were made using an alkaline phosphatase diethanolamine detection kit (Sigma-Aldrich—AP0100) following the supplier’s recommendations. Cells were detached from the discs with 400 μL of RIPA extraction and lysis buffer (Thermo Scientific—89900) and centrifuged at 13,000 × *g* for 10 minutes at 4°C. Optical density was measured spectrophotometrically at 405 nm every 3 min.

In parallel and following the same cell preparation protocol, a colorimetric assay of total proteins was performed using a Bicinchoninic Acid Protein (BCA) assay kit (Sigma-Aldrich). This is a colorimetric assay based on bicinchoninic acid, which in the presence of proteins reacts with copper to form a purple complex. A standard range was created using bovine serum albumin (Interchim—UP 36859A). Spectrophotometry at 562 nm was also used.

The ratio of ALP activity to total protein amount (BCA) thus gives the specific ALP activity.

The results are presented as percentages relative to the F-bone group (100%).

The experiment was replicated 12 times for the three bone groups after 1 day, 7 days and 14 days of culture. The analysis was performed successively on the bone disc (direct effect) and on the cell mat in the vicinity of the bone disc (indirect effect) to evaluate the effect of any release of chemical residues into the medium.

#### Evaluation of bone surface by scanning electron microscopy (SEM)

Osteoblasts morphology was studied by SEM (XL 30, Philips, The Netherlands) after 4 days of culture on the 3 different groups of bone. Two samples from each group were washed with PBS and with 0.2 M sodium cacodylate buffer pH 7.4 and fixed in 1.6*%* glutaraldehyde in cacodylate buffer overnight at 4°C (Delta microscopies, Mauressac, France).

### Statistics

Statistical analysis was performed with Excel software (Microsoft). The results are expressed as mean and standard deviation (SD). The normality of the distribution was checked by the Shapiro & Wilk test and the significance of the mean differences by Student’s *t* test when the distribution was normal, or else by Wilcoxon’s non-parametric test. The significance level chosen was *p* < 0.05.

## Results

### Assay of chemical residues in industrially processed allografts

Residues of the main four chemicals used during the cleaning process were found on the treated bone (Table 1). Residual amounts ranged from a few milligrams for urea to several grams for ethanol per kilogram of treated bone.

**Table 1:** Detailed results for chemical residues found on the chemically treated bone (CT-bone) (AUC: Area under the curve. NA: Not applicable).

	Wavelength at the maximum absorption peak on the UV spectrum (nm)	Time to peak absorption of the chemical (min)	AUC of the chemical present in the treated bone extract solution	Dilution of the pure chemical equivalent to the AUC of the treated bone extract solution	Residual quantity of chemical per kg of treated bone	Uncertainty of measurement (%)
Propanone	276	3.09	6200	1/8000	0.625 ± 0.02 g	3.3
Ethanol	220	5.25	3227	1/4850	14.03 ± 1.43 g	10.2
Urea	276	7.50	5329469	1/2350	0.0021 ± 0.0001 g	5.8
Detergent	220	1.67	13800	1/650	NA	NA

### Quantitative assessment of cell viability by the XTT method

**Fig 2:**
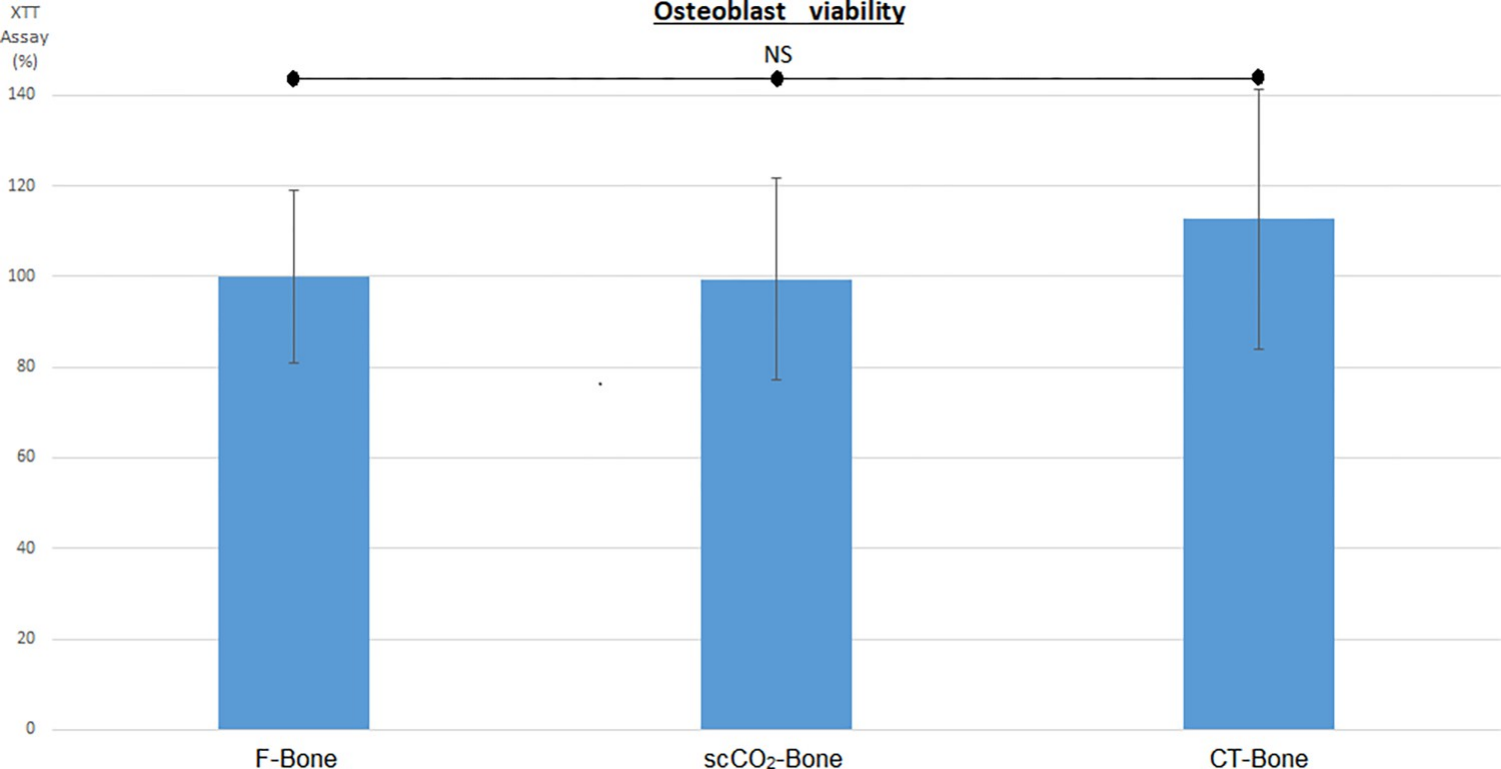
Average cell viability (XTT) of osteoblasts after 1 week of culture in the three bone groups (measurements of absorbance with a spectrophotometric reading at a wavelength of 490 nm, expressed as percentages relative to the F-bone group (100%).

After one week in culture, there was no significant difference in osteoblast cell viability between the different bone groups (F-bone: 100% (EC 23.3), scCO2-bone: 99.4% (EC 27.7), and CT-bone: 112.6% (EC 77.7), *p* > 0.1) (Fig 2).

### Evaluation of osteoblastic differentiation and mineralization by the ALP method

**Fig 3:**
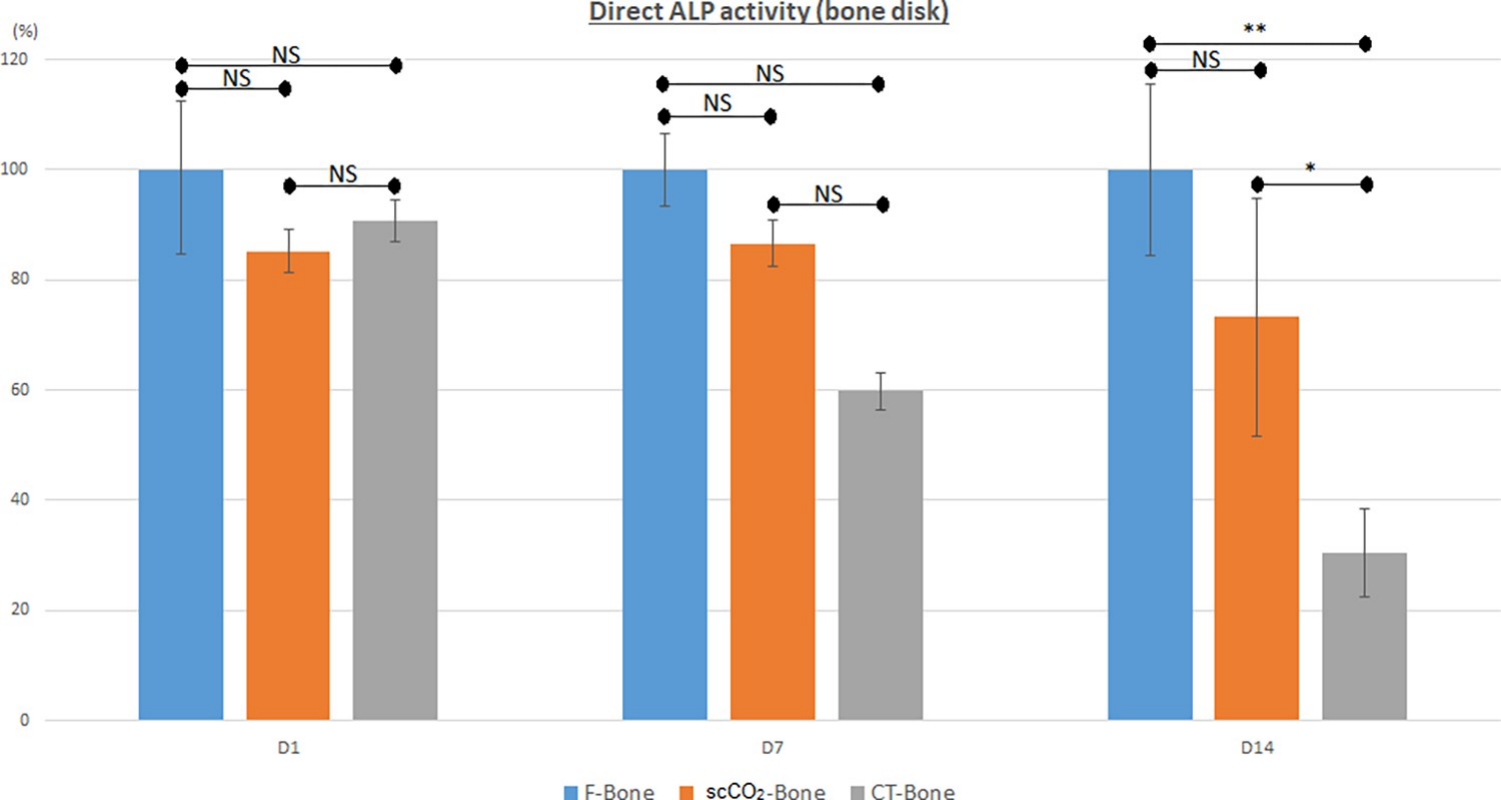
Alkaline phosphatase (ALP) activity of bone discs (direct) after 1, 7, and 14 days of culture (expressed as percentages relative to the F-bone group (100%) (NS: Not significant. *: *p* < 0.05; **: *p* < 0.01).

* Evaluation of ALP activity on the bone disc (direct effect) (Fig 3).

The ALP activities of the discs of the three groups did not differ significantly (F-bone: 100% (EC 15.4), scCO2-bone: 85.3% (EC 3.9), and CT-bone: 90.7% (EC 3.8), *p* = 0.93) after 1 day of culture.

After 7 days in culture, there was still no difference in disc ALP activity between the three bone groups, although in the CT-bone group the decrease in ALP activity was greater (F-bone: 100% (EC 6.6), scCO2-bone: 86.6% (EC 4.3) (*p* = 0.35), and CT-bone: 59.8% (EC 3.3), (*p* = 0.06).

After 14 days in culture, the ALP activity of the discs in the F-bone and scCO2-bone groups did not differ significantly (F-bone; 100% (EC 15.6), scCO2-bone: 73.3% (EC 21.7) *p* = 0.17). The ALP activity of the discs in the CT-bone group was significantly lower at 30.4% (EC 8.1), *p* = 0.01).

**Fig 4:**
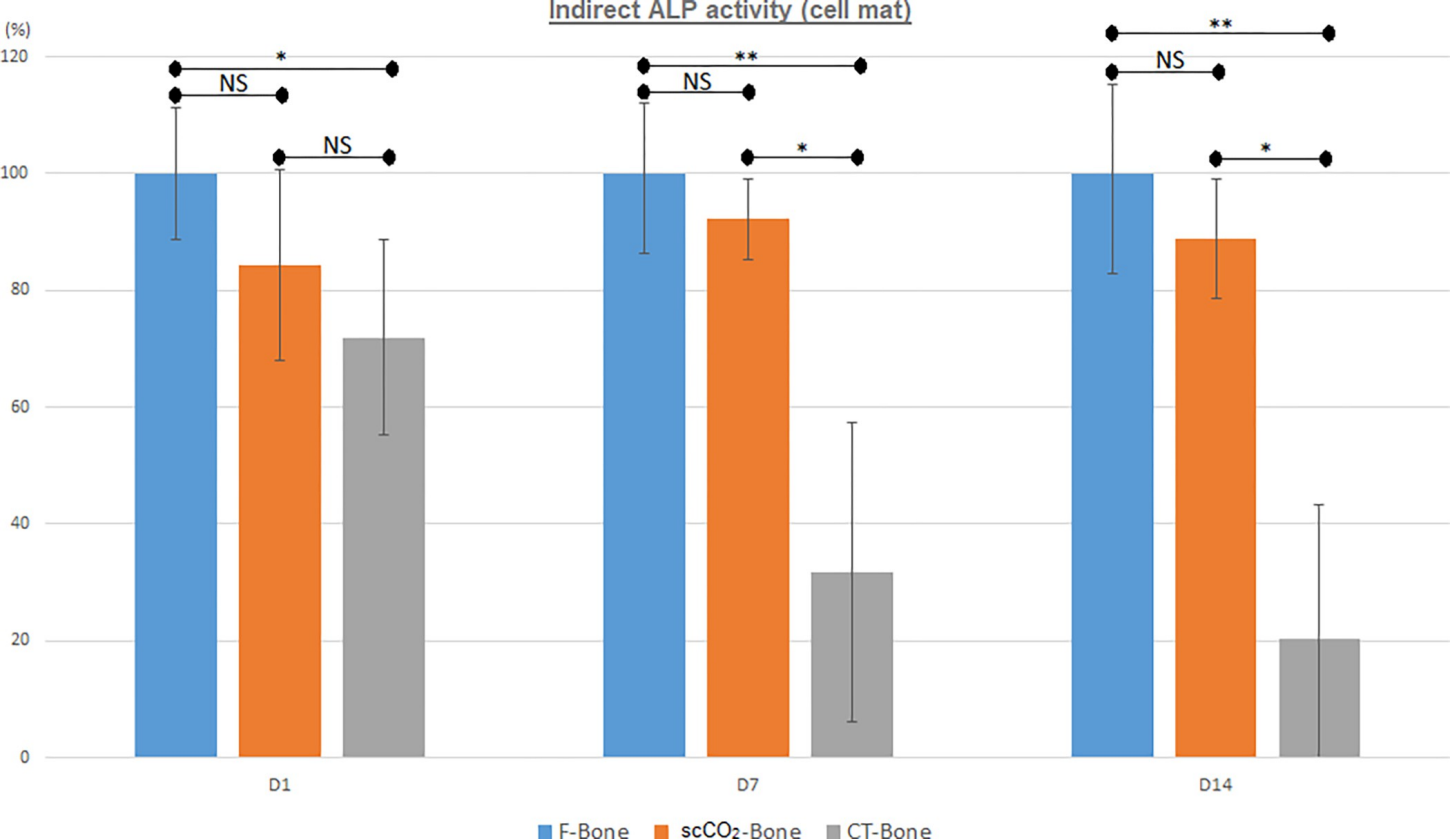
Alkaline phosphatase (ALP) activity of cell mats in the vicinity of bone discs (indirect) after 1, 7 and 14 days of culture (expressed as percentages relative to the F-bone group (100%) (NS: Not significant. *: *p* < 0.05; **: *p* < 0.01).

* Evaluation of ALP activity on the cell mat in the vicinity of the bone disc (indirect effect) (Fig 4).

After one day in culture, the ALP activity of the cell mats of the F-bone and scCO2- bone groups did not differ significantly (F-bone: 100% (EC 11.2), scCO2bone: 84.2% (EC 16.3) *p* = 0.09). The ALP activity of the mats in the CT-bone group was significantly lower at 71.9% (EC 16.8) (*p* = 0.04) than that of the F-bone group (but not the scCO2-bone group (*p* = 0.23)).

After 7 days in culture, the ALP activity of the cell mats in the F-bone and scCO2-bone groups did not differ significantly (F-bone: 100% (EC 13.8), scCO2-bone: 92.2% (EC 7.0) *p* = 0.72). The ALP activity of the mats in the CT-bone group was significantly lower at 31.7% (EC 25.7) than in the F-bone (*p* = 0.002) and scCO2-bone groups (*p* = 0.02).

After 14 days in culture, the ALP activity of the cell mats in the F-bone and scCO2-bone groups did not differ significantly (F-bone: 100% (EC 17.2), scCO2-bone: 88.7% (EC 10.2) *p* = 0.27). The ALP activity of the mats in the CT-bone group was significantly lower at 20.2% (EC 23.1) than in the F-bone (*p* = 0.001) and scCO2-bone (*p* = 0.01) groups.

### Evaluation of bone surface by scanning electron microscopy (SEM)

**Fig 5:**
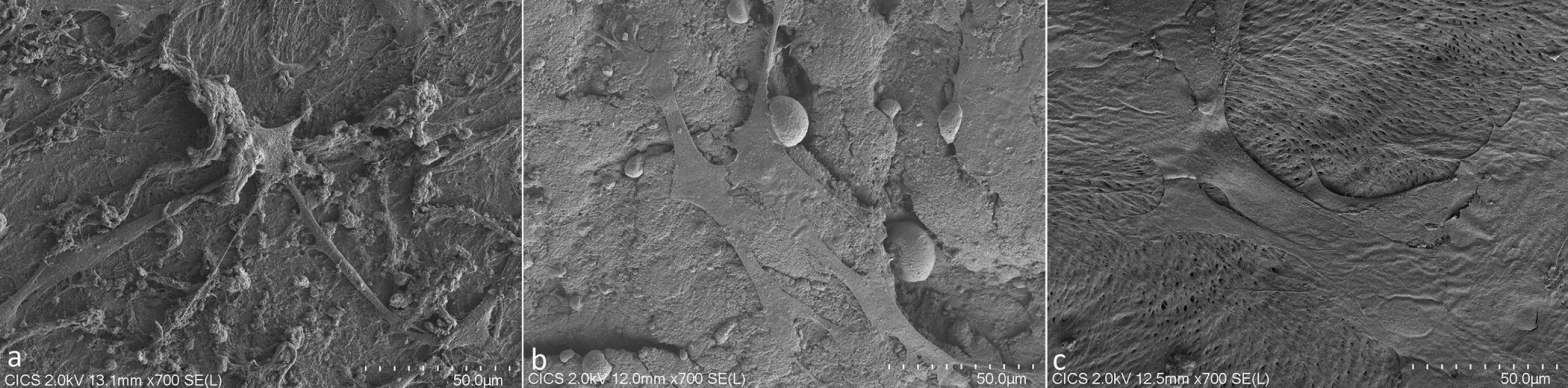
SEM images (x700 magnification) of osteoblasts on the 3 different bones after 4 days of culture (a: « F-bone », b: « scCO2-bone », c: « CT-bone »).

**Fig 6:**
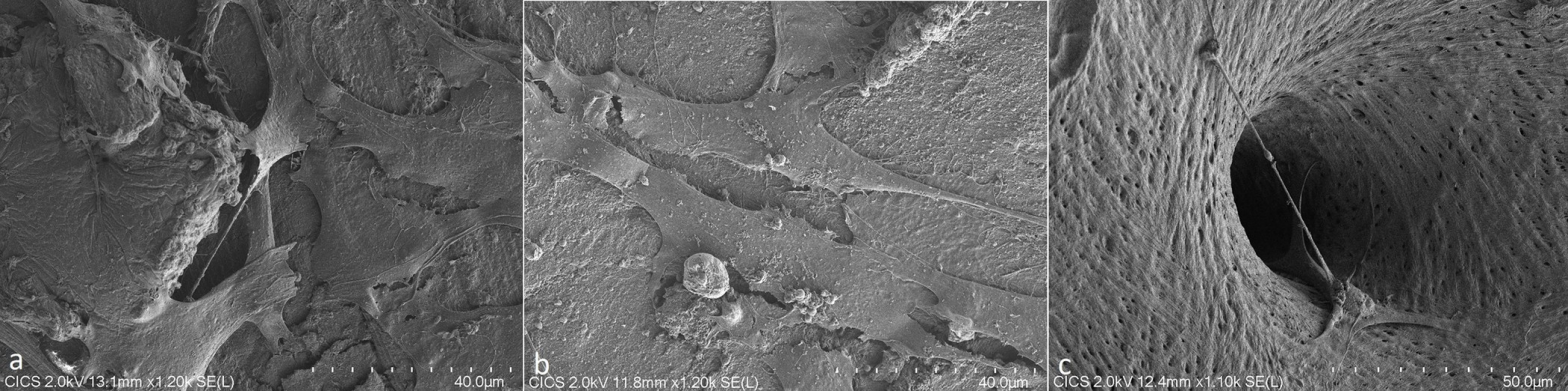
SEM images (x1200 magnification) of osteoblasts on the 3 different bones after 4 days of culture (a: « F-bone », b: « scCO2-bone », c: « CT-bone »).

Osteoblasts appeared as large flattened and elongated cells (Fig 5). Cells were at confluence in some areas. Cytoplasmic extensions were visible as well as on plane and porous surfaces (Fig 6). They were no obvious difference between the 3 groups of bones.

## Discussion

The results of this study support our initial hypothesis that after a cleaning process using chemicals, residues persist on the bone allograft and cause an *in vitro* decrease in the differentiation capacity of osteoblasts, though without a decrease in their viability. These chemicals therefore lead to a decreased biocompatibility of the allografts.

The main role of a bone substitute is to restore lost substance and to serve as a framework for the "regeneration" of bone tissue. Irrespective of how they are preserved, allografts must therefore be highly biocompatible to favor colonization by host cells.

The goal of this study is to evaluate the impact of bone processing with and without chemicals on biocompatibility. Consequently, a study with 3 groups was designed.

Frozen-bone (without any treatment or preservative) represent the historical preservation mode. It is the control group.

The scCO2-bone is a "model" of bone processed with no chemicals. scCO2 has been the most widely used supercritical fluid in food and scientific industries for 40 years because it acts as a totally neutral and non-toxic solvent [15]. The results of cell viability and osteoblastic differentiation and mineralization in this group showed no significant difference from the reference F-bone group. This confirms that a cleaning treatment without potentially toxic chemicals does not affect the biocompatibility of the bone.

The”CT-bone” is a commercially available treated bone allograft. It represents a traditional and validated method of treatment with chemicals. The various chemicals used in the additional treatments of allografts are all classified as presenting health hazards by European regulation No. 272/2008, known as CLP (Classification, Labelling, Packaging) [16]. In our study, residues of the 4 chemicals used during cleaning were found at concentrations ranging from 21.25 ± 1.23 mg for urea to 14.03 ± 1.43 g for ethanol (per kilogram of bone treated).

These are minimum concentrations and although they can be considered low in absolute terms, they nevertheless induce an *in vitro* negative effect on osteoblast culture. The direct and indirect ALP activities of the osteoblast cultures (osteoblastic differentiation on the bone disc and on the cell mat in the vicinity of the bone disc) were significantly lower after 7 and 14 days in the CT-bone group. However, there was no significant difference in cell viability (XTT).

To support these results, an additional osteoblast culture experiment was performed with conditioned culture media prepared from 10 g of finely ground cancellous bone (from each of the F-, scCO2- and C-bone groups) steeped in 20 mL of medium for 48 h with agitation (S1 Appendix). Under these conditions, with a much higher concentration of bone in contact with the culture medium, cell viability (XTT) and osteoblastic activity (ALP) were significantly decreased by about 50% in the CT-bone group (*p* = 0.03 and 0.001, respectively) compared to the other two bone groups (F and scCO2).

Under different experimental conditions (bones steeped in separate baths of chemicals), Dumas et al. [17] obtained results in line with ours. After one week of culture, they noted a slight decrease in cell proliferation, but more especially in ALP activity, of 40–60% when hydrogen peroxide and sodium hydroxide were used (either separately or combined) but not with ethanol. The explanation might be that these chemicals altered the organic and inorganic phases of the bone, causing lysis of the extracellular matrix proteins and a decrease in the presence of growth factors regulating the recruitment and activity of osteoblasts and osteoclasts, such as TGF, osteopontin, osteonectin, or bone morphogenic proteins [18–21]. In our study, the decrease in direct ALP activity (on the bone disc) in the CT-bone group supports this explanation. The decreased indirect ALP activity (on the cell mat in the vicinity of the bone disc) in the CT-bone group and the results of cultures in conditioned media (S1 Appendix) more probably reflect another phenomenon, namely the release of chemicals present on the surface of the bone with direct toxicity for the osteoblasts. Rasch et al. [22] focused on the effect of chemically versus sonication-based processing method on decellularization and biocompatibility. Finally, they found similar decrease of biocompatibility with these two processing methods compared to control. Nonetheless, chemically processed grafts were washed three times for 2 hours with PBS on an orbital shaker before culture, which probably removed chemical residues.

This study has some limitations. Concerning the extraction method, a “simulated extraction” was chosen according to the biological evaluation of medical devices ISO 10993–12. It is performed in order to estimate the type and quantity of substances assumed to be released by a medical device during its clinical use (different from an exaggerated or exhausted extraction). Consequently, this method may not have extracted all the residues of the chemicals present on the bone or causing their degradation. The residual concentrations found are therefore minimum levels. It would also have been of interest to look for degradation products of these chemicals because these can also present some toxicity. The standard deviations of the results of the osteoblast cultures on the bone discs probably reflect inter-individual differences in the bones of healthy donors. This was controlled by repeating the tests (12 times) with bone from four different donors. Finally, these experimental findings must be viewed in the light of clinical studies on the use of processed bone allografts that report low clinical and radiological failure rates (but osseointegration is rarely studied histologically) [23–25].

## Conclusion

The presence on treated bone allografts of residues of chemicals used during the cleaning process caused an *in vitro* decrease in their biocompatibility. This finding justifies changes in tissue cleaning practices, limiting or replacing these chemicals to favor biocompatibility and ensure a high level of safety with respect to microbiological risk.

## Supporting information

S1 Fig (TIF)

S2 Fig (TIF)

S1 Appendix (DOCX)

S1 File Individual level data underlying Table 1 and Figs 2–4. (DOCX)
