# Expression of Concern: *In vitro* osteoblast activity is decreased by residues of chemicals used in the cleaning and viral inactivation process of bone allografts

**DOI:** 10.1371/journal.pone.0298054

**Published:** 2024-02-01

**Authors:** The PLOS ONE Editors

After editorial follow up, at the time of publication of this notice, this article [1] was republished to remove proprietary information. The *PLOS ONE* Editors issue this Expression of Concern to notify readers that the updated article does not report adequate methodological information for the study to be reproducible.

At the time of republication, the additional issues detailed below were also addressed in the revised text. Please download the article again to view the correct version.

Additional issues:

The Competing Interests statement in the originally published article declared that no competing interests exist. The Competing Interests statement has been revised to state: Authors GV, RE, SD, and SB have made a Declaration of Invention regarding the specific cleaning process using supercritical carbon dioxide described in this study. This does not alter our adherence to *PLOS ONE* policies on sharing data and materials.

There were errors in the reporting of the Materials and methods section in the originally published article. The first sentence of the Materials and methods subsection Origin and preservation of allograft bone incorrectly reported that all bone tissues were obtained from Multi-Organ Tissue Harvesting procedures. The correct text in the republished article is:

Bone tissues (cancellous and cortical) used in the F-bone group and the scCO_2_-bone group came from femurs obtained in four Multi-Organ Tissue Harvesting (MOTH) procedures. Samples used in the CT-bone group were from living female donors receiving hip arthroplasty and manufactured as demi-tete products for therapeutic use; residues (the cortical part of the neck) not used for the care of the patient, considered surgical waste, were collected for use in this study.

A member of the Editorial Board has reviewed the revised methodological information and advised that this does not affect the support for the reported results and conclusions.

The revised methods text also clarifies the relative timing of the bone preservation and cutting, with the following revised text: Cortical bone fragments were cut as discs 10 mm in diameter and 3 mm thick after the preservation procedures were carried out.

Underlying data for the article have been provided as S1 File in the Supporting Information in the republished article. Underlying individual-level data for S1 Fig and S2 Fig remain available upon request from the corresponding author.
