# Supplementary figures and images for: In vitro osteoblast activity is decreased by residues of chemicals used in the cleaning and viral inactivation process of bone allografts
Source: PLoS One. 2022 Oct 10;17(10):e0275480. doi: 10.1371/journal.pone.0275480 (PMC9550034; doi:10.1371/journal.pone.0275480)

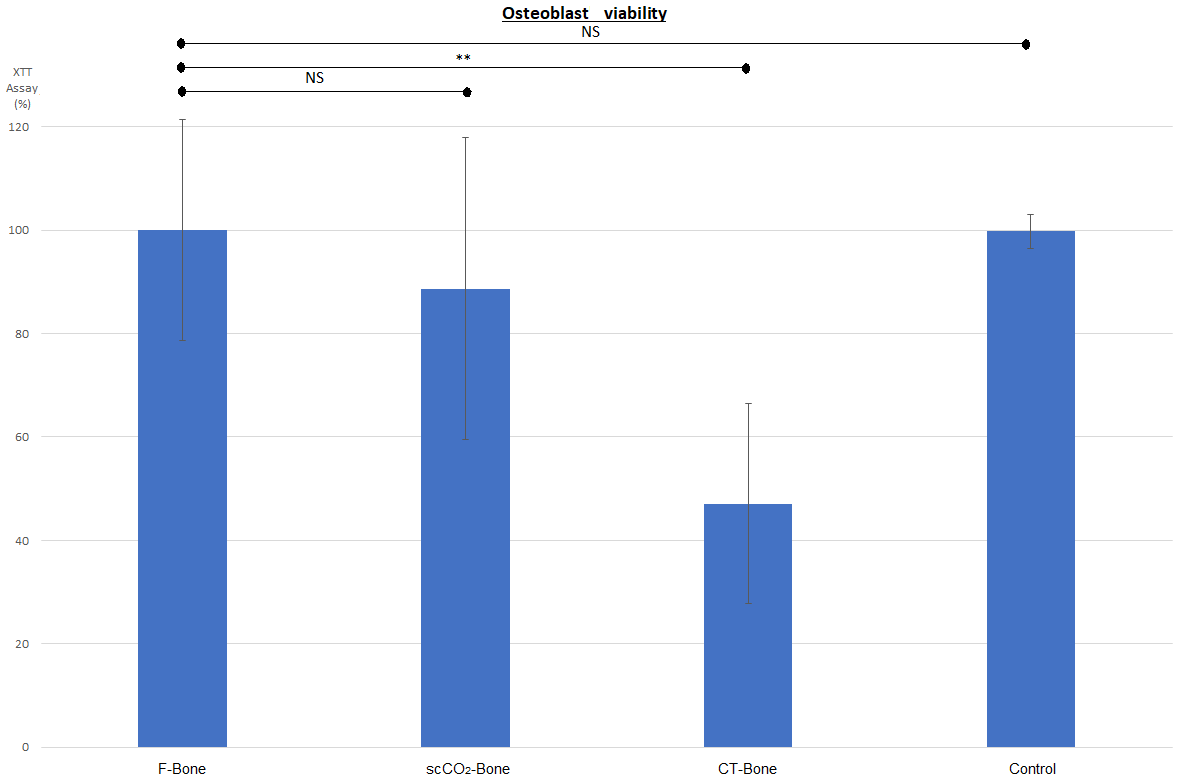

Supplement: S1 Fig — (TIF) [file pone.0275480.s001.tif]

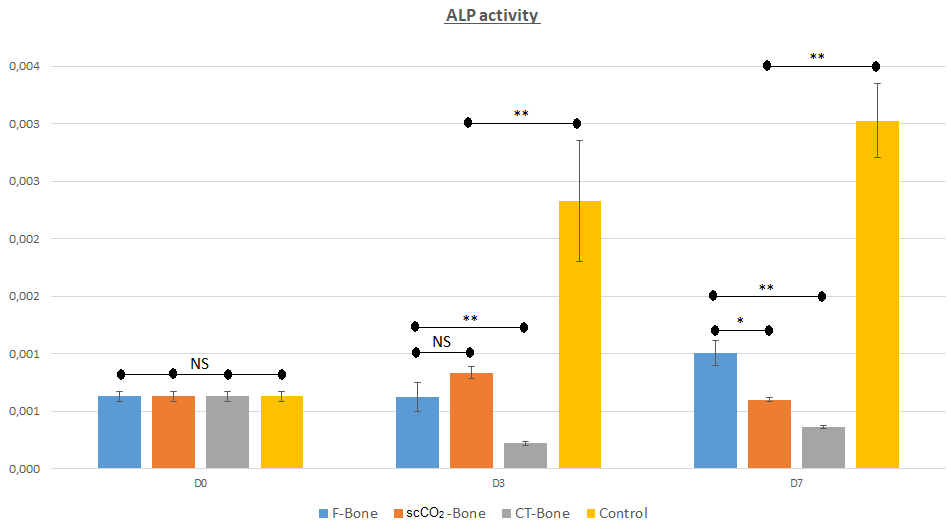

Supplement: S2 Fig — (TIF) [file pone.0275480.s002.tif]
