# Supplementary material for: In vitro osteoblast activity is decreased by residues of chemicals used in the cleaning and viral inactivation process of bone allografts
Source: PLoS One. 2022 Oct 10;17(10):e0275480. doi: 10.1371/journal.pone.0275480 (PMC9550034; doi:10.1371/journal.pone.0275480)
Supplement: S1 Appendix — (DOCX) [file pone.0275480.s003.docx]

**Appendix 1**

**Materials and Methods**

***Preparation of conditioned media and culture of primary rat osteoblast cells***

Three conditioned culture media were made from the three different bone groups (F-, scCO2-, and CT-bones). From each bone type, 10 g of finely ground cancellous bone was steeped in 20 ml of α-MEM culture medium supplemented with 10% fetal bovine serum (FBS) and 1% p/s for 48 h.

In 12-well plates equipped with 8 µm membranes (Falcon) were seeded 35,000 primary osteoblasts (10,000 cells/cm²) per well, and the whole covered with 2 mL of culture medium. Plates were incubated at 37 °C and 5% CO_2_, without shaking. After 48 h of culture, the culture medium was removed.

In the wells containing the osteoblasts was then deposited 2 ml of conditioned culture medium from either the F-bone, scCO2-bone, or CT-bone group. A control with standard culture medium was run in parallel. The plates were then incubated at 37 °C and 5% CO_2_, without shaking. The conditioned culture medium was changed three times a week.

Cell viability (XTT) was assessed after 7 days of culture. Evaluation of osteoblastic differentiation (ALP) was made after 3 and 7 days of culture. The experiments were performed in triplicate with one control in each case.

**Results**

***Cell viability of primary rat osteoblast cultures with conditioned culture media (Fig. 1)***

After one week in culture, the cell viability of the F-bone and scCO2-bone groups did not differ significantly and was comparable to the control (F-bone: 100% (EC 21.3), scCO2-bone: 88.7% (EC 29.2); *p* > 0.1). Viability in the CT-bone group was significantly lower than in the other groups (47.1% (EC 19.4); *p* = 0.03).


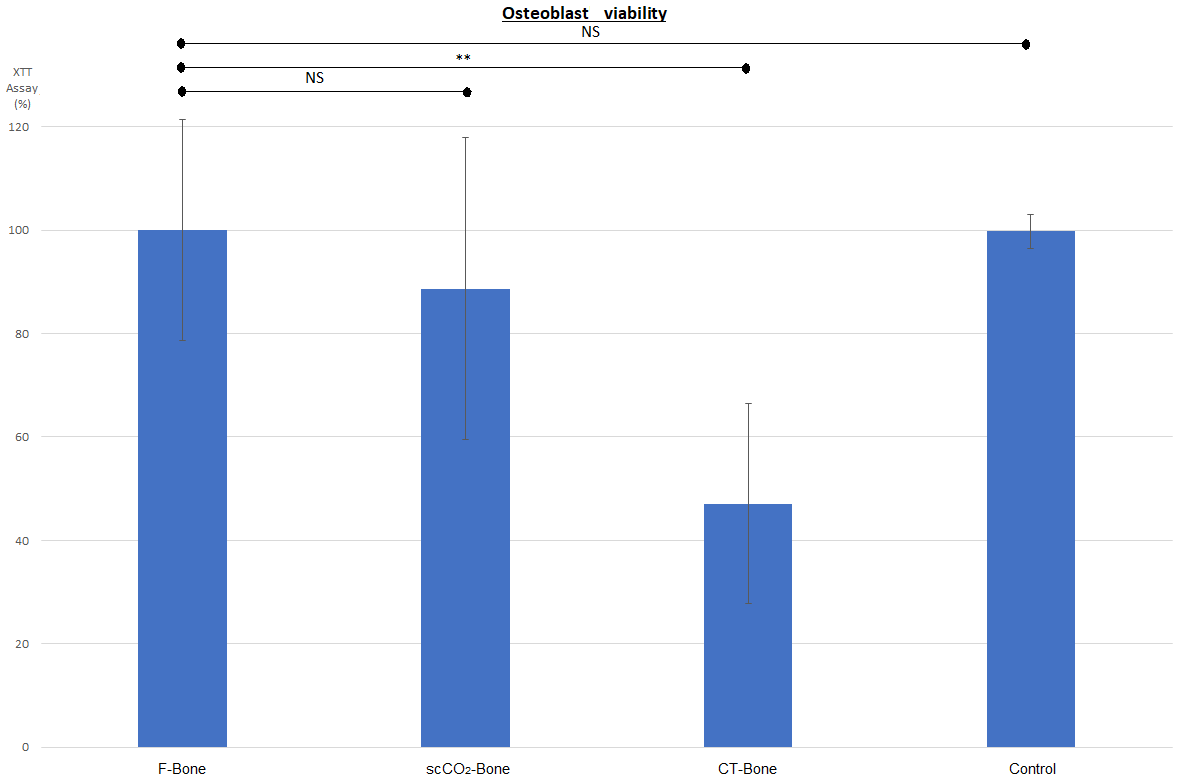


Figure 1. Average cell viability of osteoblasts after culture in conditioned media (expressed as percentage relative to the F-bone group (100%) (NS: not significant. *: *p* < 0.05; **: *p* < 0.01).

***Evaluation of ALP activity in conditioned media*** (Fig. 2)

The ALP activities of the four groups were equal at D0.

The ALP activity in the control group (standard culture medium) increased significantly at D3 (+250%) and at D7 (+400%) (*p* < 0.001).

The ALP activity in the F-bone conditioned group was equivalent between D0 and D3 (+0%, *p* = 0.33) and then significantly increased at D7 (+50%, *p* = 0.02).

The ALP activity in the scCO2-bone conditioned group increased significantly between D0 and D3 (+30%, *p* = 0.04) and then decreased between D3 and D7 to become equivalent to that of D0 (−0%, *p* = 0.45).

The ALP activity of the CT-bone conditioned group was significantly decreased at D3 (−60%) and at D7 (−45%) (*p* = 0.001). The ALP activity of this group was significantly lower than those of the C-bone and scCO2-bone conditioned groups  (*p* < 0.01) at each time point.


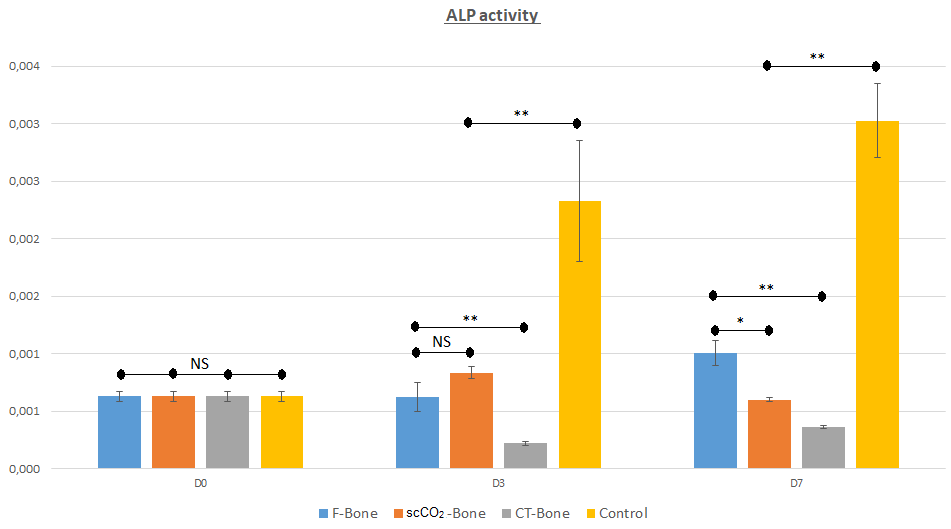


Figure 2. ALP activity after culture in conditioned medium (F-bone, scCO2-bone, CT-bone) and standard culture medium (control) at D0, D3 and D7 (expressed as ALP units) (NS: not significant. *: *p* < 0.05; **: *p* < 0.01).
