# Supplementary material for: In vitro osteoblast activity is decreased by residues of chemicals used in the cleaning and viral inactivation process of bone allografts
Source: PLoS One. 2022 Oct 10;17(10):e0275480. doi: 10.1371/journal.pone.0275480 (PMC9550034; doi:10.1371/journal.pone.0275480)
Supplement: S1 File — (DOCX) [file pone.0275480.s004.docx]

Table 1 :

propanone


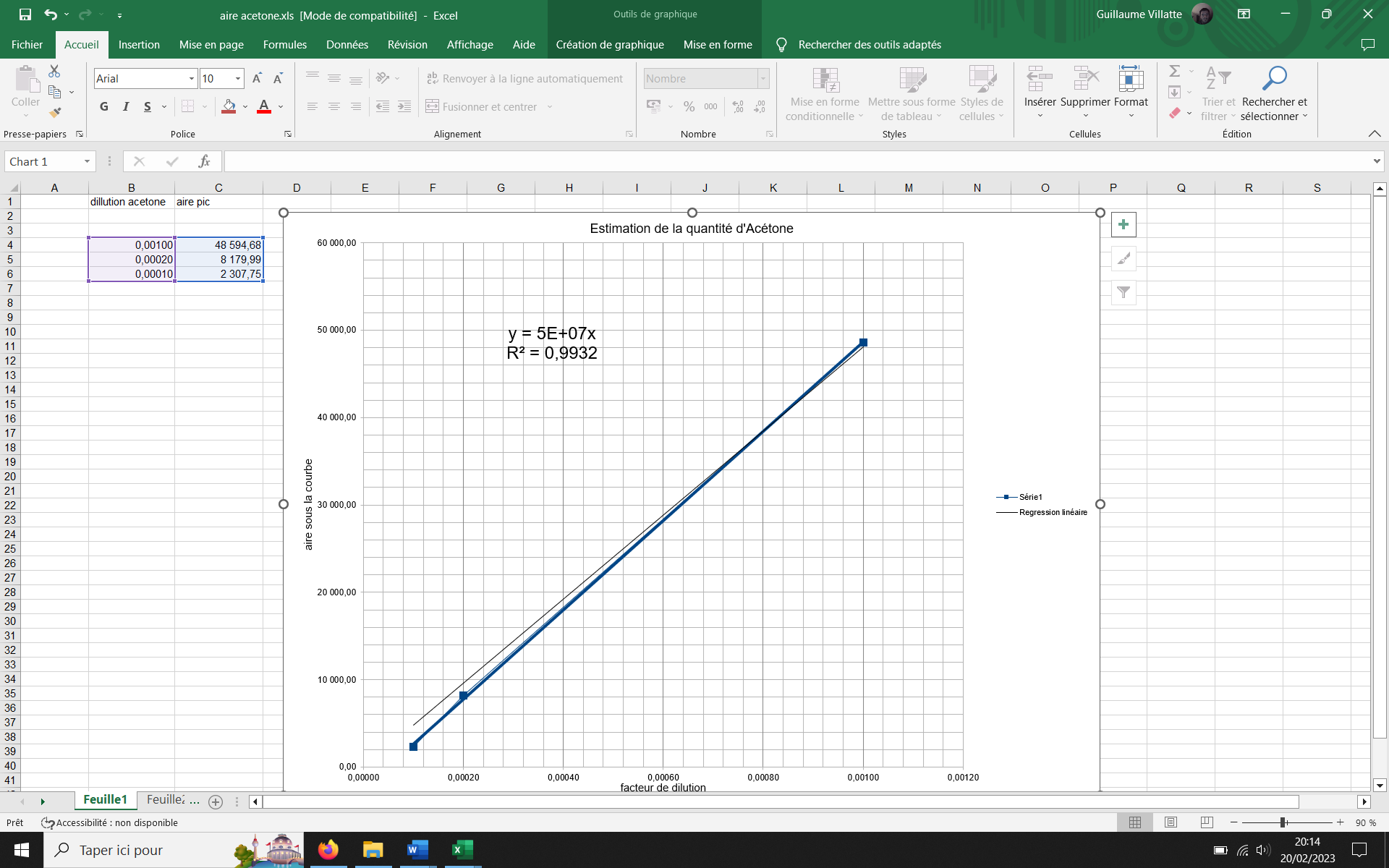


Ethanol


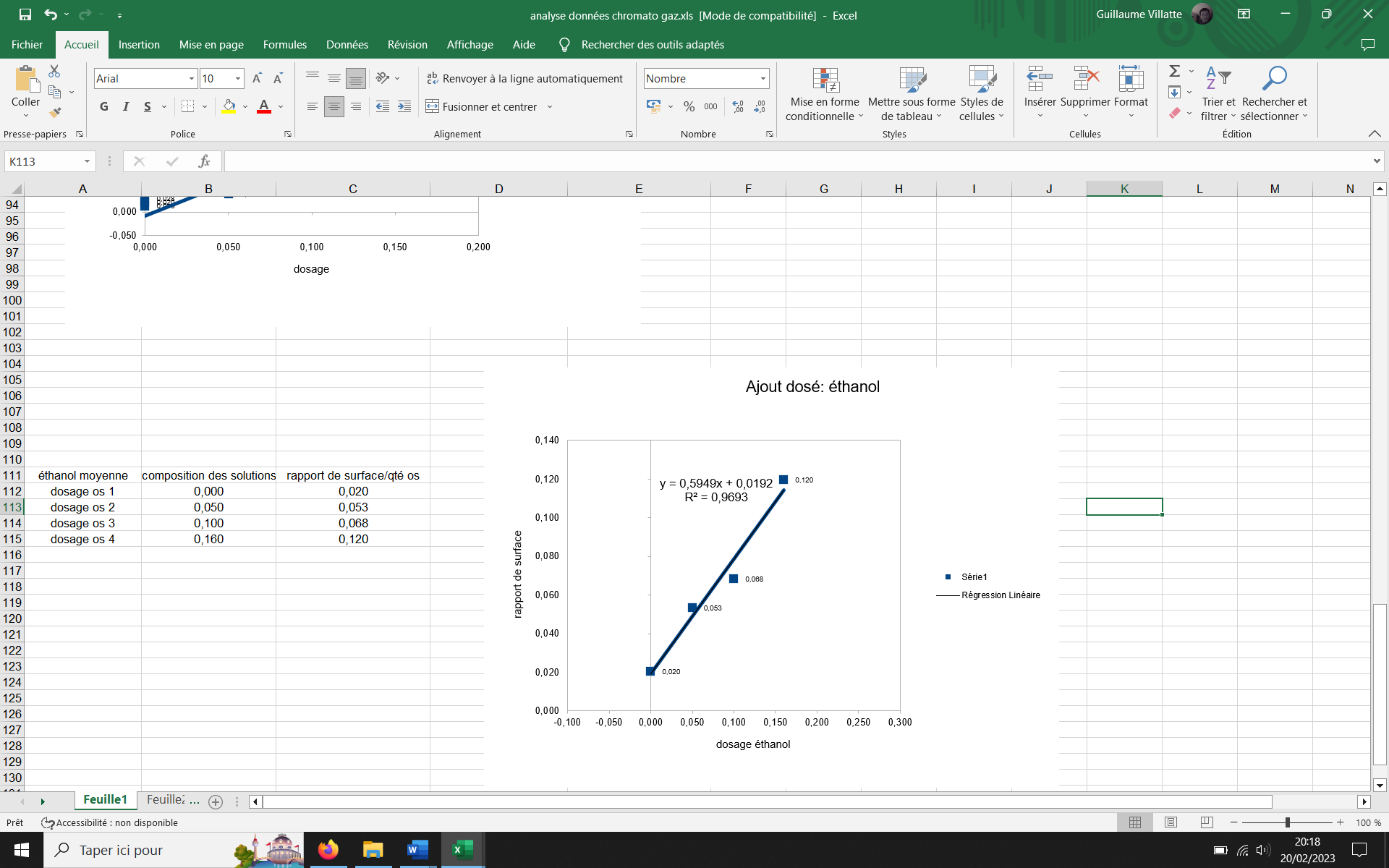


Urea


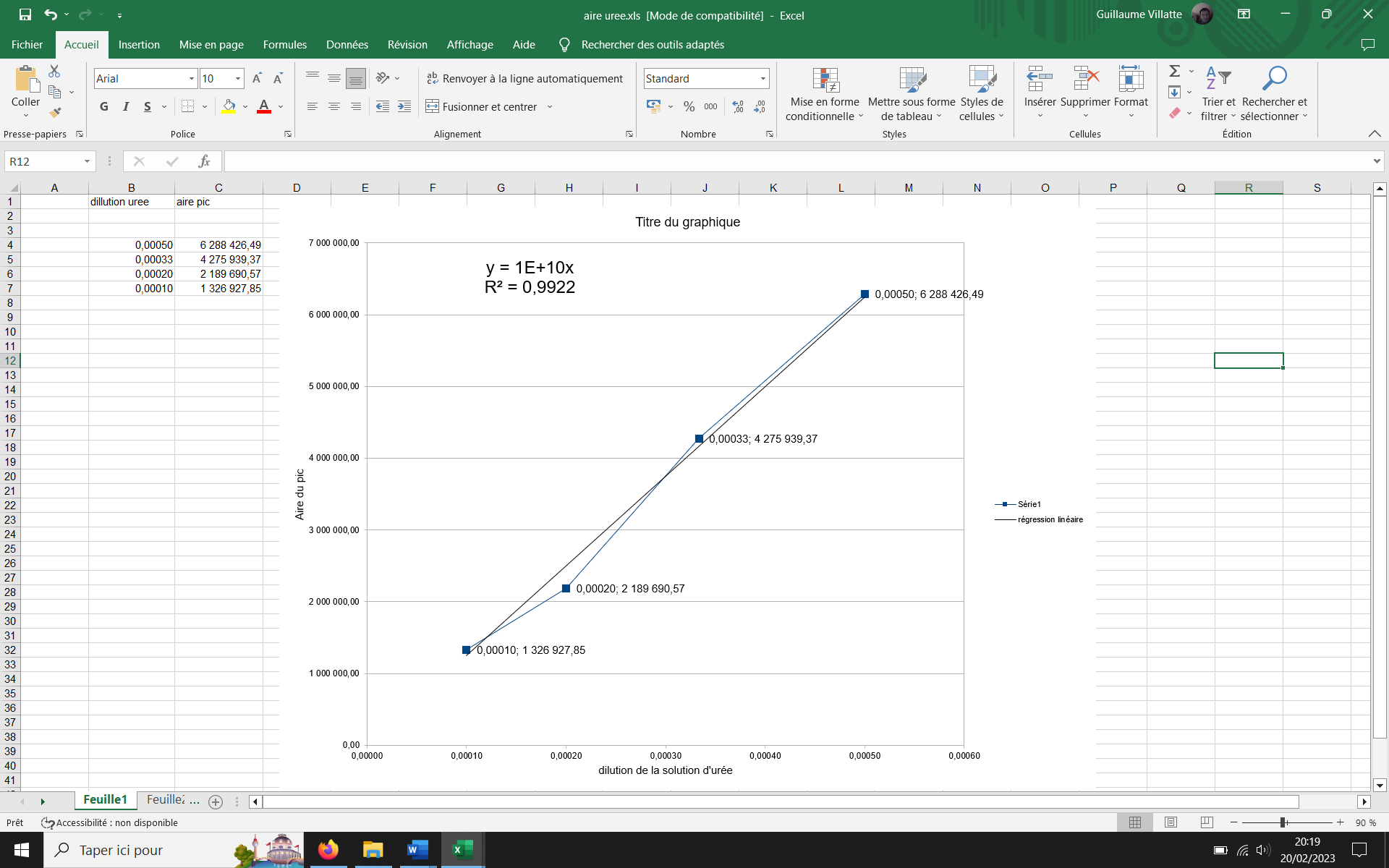


Detergent


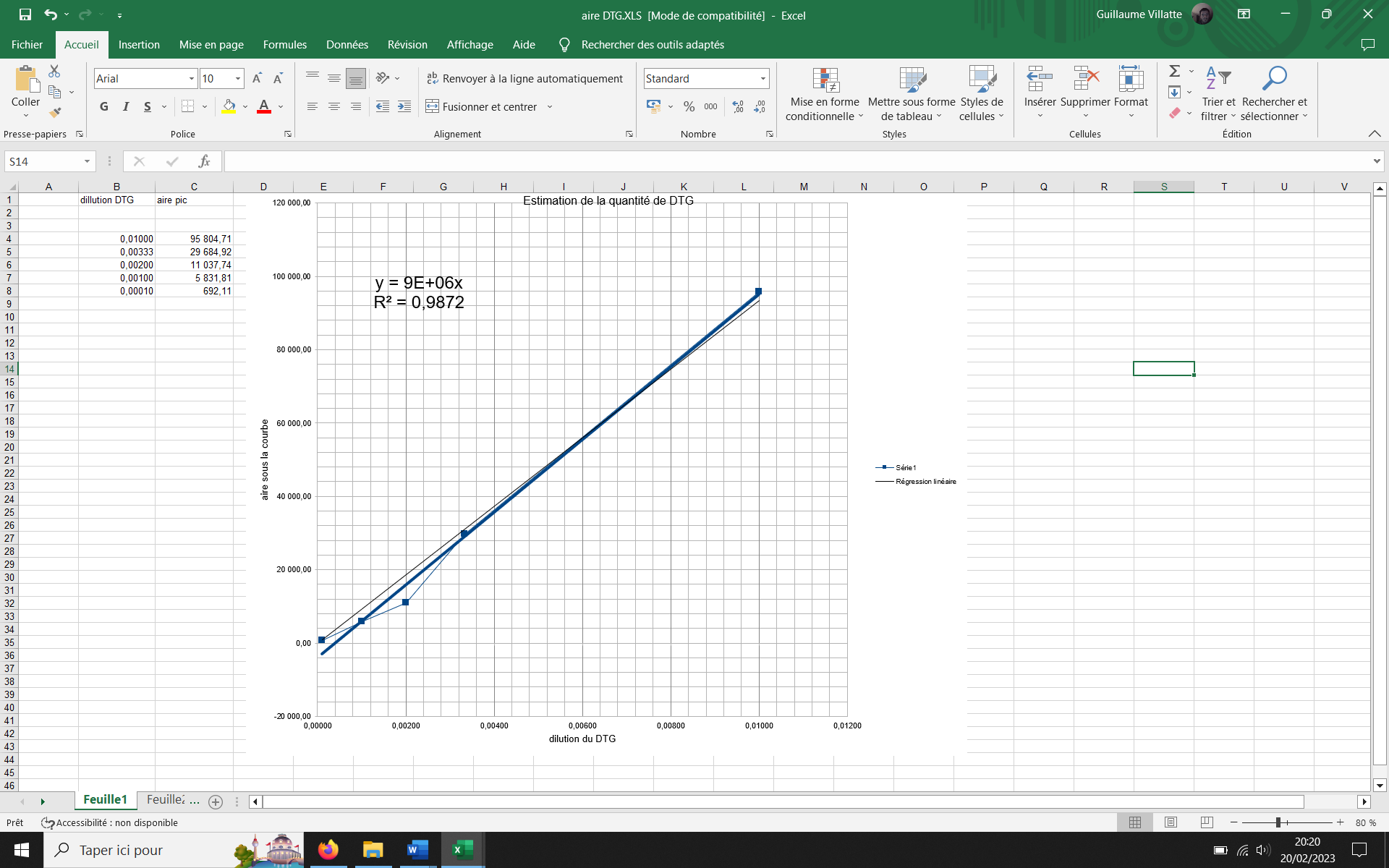


-Fig 2 :

|  | F-bone | scCO2-bone | CT-bone |
| --- | --- | --- | --- |
|  | 137,751561 | 141,568355 | 90,5621096 |
|  | 100,277585 | 107,21721 | -19,0839695 |
|  | 45,4545455 | 78,0707842 | 143,650243 |
|  | 98,5426787 | 56,5579459 | 108,605135 |
|  | 117,973629 | 148,854962 | 251,561416 |
|  | 95,5414013 | 106,46154 | 100,277585 |
|  | 93,0955414 | 116,484076 | 91,8821096 |
|  | 109,382166 | 109,643312 | 265,581416 |
|  | 114,286624 | 78,5832484 | 117,820243 |
|  | 99,8216561 | 64,1656051 | 88,9951353 |
|  | 90,8726115 | 94,4012739 | 45,2839695 |
|  | 97,001345 | 90,8726115 | 65,597585 |
| Mean | 100,000112 | 99,4067437 | 112,561081 |
| ec | 21,2186453 | 27,6540877 | 77,6985098 |

-Fig 3 :

|  | D1 | D7 | D14 |
| --- | --- | --- | --- |
| F-Bone | 112,442127 | 94,1040342 | 108,99 |
|  | 70,0179469 | 110,520264 | 81,33 |
|  | 97,5778732 | 92,3757022 | 106,67 |
|  | 127,782753 | 92,7122052 | 111,58 |
|  | 86,2172469 | 106,109677 | 92,46 |
|  | 110,787753 | 95,1781176 | 115,96 |
|  | 88,255379 | 108,888505 | 127,2488526 |
|  | 108,99777 | 104,721321 | 81,06579945 |
|  | 90,9268509 | 91,0901743 | 91,68534794 |
|  | 98,855379 | 99,9854 | 108,999865 |
|  | 114,91777 | 103,8769 | 99,000100100 |
|  | 92,9268509 | 100,009865 | 75,11112854 |
| MEAN | 99,975475 | 99,9643471 | 100,00925780 |
| EC | 15,3592014 | 6,67868593 | 15,57937862 |
| scCO2-Bone | 83,0981219 | 98,5089583 | 81,42630932 |
|  | 84,1362088 | 87,2655389 | 86,33628696 |
|  | 90,7383947 | 83,1316935 | 105,0148032 |
|  | 80,9437834 | 87,358658 | 33,52924348 |
|  | 87,5874898 | 82,2424781 | 101,5684563 |
|  | 83,7656595 | 85,5714074 | 62,91511125 |
|  | 89,7476544 | 81,9687087 | 84,19245726 |
|  | 81,6659393 | 88,1512788 | 74,78244876 |
|  | 82,5641114 | 85,5160744 | 73,66290597 |
|  | 88,0465896 | 86,1334686 | 74,999653 |
|  | 79,9865666 | 87,0008652 | 39,000869 |
|  | 91,0047566 | 86,6523456 | 61,0000986 |
| MEAN | 85,273773 | 86,625123 | 73,20238692 |
| EC | 3,94424418 | 4,26743132 | 21,70450823 |
| CT-Bone | 94,90373 | 59,8512919 | 29,84683574 |
|  | 86,889065 | 61,6653691 | 41,62877826 |
|  | 91,9998765 | 66,9999699 | 33,3289428 |
|  | 91,9009085 | 60,5692435 | 25,51049843 |
|  | 84,1011235 | 54,6699357 | 26,0043967 |
|  | 96,2998354 | 55,1075885 | 26,08102744 |
|  | 92,45586 | 62,999886 | 38,087534 |
|  | 88,8596062 | 56,89954 | 21,83674 |
|  | 90,734567 | 58,45678 | 37,9864 |
|  | 90,1234123 | 60,674942 | 23,0125432 |
|  | 85,0264145 | 59,9888234 | 43,0008235 |
|  | 94,9986438 | 59,876592 | 18,1569222 |
| MEAN | 90,69 | 59,8133 | 30,37 |
| EC | 3,8187237 | 3,30363318 | 8,076121917 |

-Fig 4 :

|  | D1 | D7 | D14 |
| --- | --- | --- | --- |
| F-Bone | 77,81957663 | 79,8119033 | 102,52 |
|  | 109,8717899 | 117,707757 | 84,72 |
|  | 92,30863347 | 93,4803397 | 111,76 |
|  | 121,1385561 | 88,8503669 | 113,19 |
|  | 94,29917241 | 110,592901 | 72,41 |
|  | 104,5622715 | 109,056732 | 114,40 |
|  | 89,11824781 | 114,366393 | 111,390398 |
|  | 111,4503146 | 93,2424337 | 66,4604219 |
|  | 99,43143758 | 92,3911731 | 124,94918 |
|  | 105,24746 | 106,00976 | 99,999923 |
|  | 94,863645 | 95,88899 | 100,111175 |
|  | 99,99886749 | 98,687899 | 98,1225689 |
| MEAN | 100,009 | 100,007221 | 100,002806 |
| EC | 11,2385026 | 11,2167148 | 17,1985846 |
| scCO2-Bone | 132,0807897 | 95,3906241 | 76,3473863 |
|  | 93,80610543 | 79,5315708 | 77,3868358 |
|  | 72,86532029 | 87,3819972 | 90,8768971 |
|  | 75,16748019 | 83,5664297 | 95,8523518 |
|  | 76,48416018 | 88,5673745 | 99,2276185 |
|  | 77,89488823 | 93,4381735 | 67,7373548 |
|  | 82,2306312 | 96,6280035 | 95,5240805 |
|  | 74,34772418 | 104,574676 | 102,436314 |
|  | 73,31178493 | 100,123943 | 93,0713642 |
|  | 85,176001 | 94,0009678 | 90,72056 |
|  | 82,9997897 | 89,000023 | 86,000356 |
|  | 84,59998679 | 93,80894 | 89,649878 |
| MEAN | 84,24705516 | 92,1677269 | 88,7359164 |
| EC | 16,28223451 | 6,97227055 | 10,2396456 |
| CT-Bone | 87,6423226 | 42,8866459 | 57,4765963 |
|  | 76,87719067 | 81,8134068 | 23,7968671 |
|  | 98,96022359 | 24,2466245 | 23,0342736 |
|  | 59,46835646 | 25,311442 | 4,03631825 |
|  | 44,57534699 | 25,4244396 | 3,83579208 |
|  | 48,67043666 | 21,1600422 | 4,16767772 |
|  | 77,6423226 | 27,0845874 | 67,759348 |
|  | 56,87719067 | 86,8314068 | 43,7968671 |
|  | 89,26022359 | 9,24244632 | 8,10423645 |
|  | 84,46835646 | 10,311442 | 1,06382528 |
|  | 75,57534699 | 15,4244396 | 2,32920779 |
|  | 63,37043666 | 11,1422037 | 3,41677226 |
| MEAN | 71,95 | 31,7399 | 20,2348 |
| EC | 16,81065071 | 25,6962497 | 23,085539 |
